# The knowledge and attitudes of general practitioners to the assessment and management of pain in people with dementia

**DOI:** 10.1186/s12875-018-0853-z

**Published:** 2018-10-10

**Authors:** Aisling A Jennings, Maura Linehan, Tony Foley

**Affiliations:** Department of General Practice, Western Gateway Building, University College Cork, Cork, Ireland

## Abstract

**Background:**

Pain in people with dementia is underdiagnosed and undertreated. General practitioners (GPs) play a pivotal role in dementia care but their perspectives on pain in people with dementia remains under-researched. The aim of this study was to explore GPs’ knowledge and attitudes towards pain assessment and management in people with dementia.

**Methods:**

This was a descriptive cross-sectional study. A questionnaire was adapted from a previous study and piloted with 5 GPs. The questionnaire was posted to a census sample of all GPs in Cork city and county in the southern region of Ireland. The questionnaire collected demographic information, responses to a series of Likert-type statements assessing GPs’ knowledge and attitudes, and provided an opportunity for the GP to give qualitative feedback on their experiences of managing pain in dementia. SPSS v25 was used for statistical analysis. Qualitative responses were thematically analysed.

**Results:**

Of the 320 questionnaires posted, 157 completed questionnaires were returned (response rate of 49%). The sample was representative of GPs nationally in terms of years in GP practice and practice location. Over two-thirds (108/157) of respondents had a nursing home commitment. Only 10% of respondents (16/157) were aware of any dementia-specific pain assessment tools. The larger the nursing home commitment of the GP the more likely they were to be familiar with these tools (*p* = 0.048). The majority of respondents (113/157) believed people with dementia could not self-report pain. Respondents were uncertain about the safety of using opioid medications to treat pain in people with dementia with only 51.6% agreeing that they were safe. The qualitative comments highlighted the importance the GPs placed on surrogate reports of pain, GPs’ uncertainty regarding the value of formal pain assessment tools and the challenges caused by under-resourcing in general practice.

**Conclusion:**

This study has highlighted aspects of pain assessment and management in dementia that GPs find challenging. Guidance on pain assessment and management in people with dementia do not appear to be translating into clinical practice. The findings will inform educational interventions being developed by our research team as part of the implementation of the Irish national dementia strategy.

The knowledge and attitudes of general practitioners to the assessment and management of pain in people with dementia.

**Electronic supplementary material:**

The online version of this article (10.1186/s12875-018-0853-z) contains supplementary material, which is available to authorized users.

## Background

The global prevalence of dementia is increasing. In 2013 it was estimated that 44 million people worldwide were living with dementia and this figure is expected to reach 75 million in 2030 and 135 million by 2050 [1]. Although these estimates may be reduced by improvements in population health, such as reductions in smoking and hypertension, current evidence suggest that only 10% of the expected rise in incidence will be avoided by improvements in these disease control measures [2]. A second major health issue facing the older population is that of chronic pain. The prevalence of pain is strongly correlated with increasing age [3, 4]. People with dementia appear to have a significantly increased risk of pain [5], with up to half of people with dementia estimated to be living with chronic pain [6–8]. In one study of nursing home residents the prevalence of chronic pain in residents with dementia was almost double that of residents without dementia [9]. Similarly, in the community setting, pain is more prevalent in people with dementia than in people without dementia [5, 7, 10]. This increased prevalence is related, at least in part, to the significantly higher burden of co-morbid physical disease in people with dementia [11, 12] which contributes to increased musculoskeletal pain [13, 14], orofacial pain [15] and neuropathic pain [16]. However, pain in people with dementia is often underdiagnosed, underestimated and undertreated [9, 17, 18].

The negative impact of undiagnosed, untreated pain in dementia is substantial. In a person with dementia untreated pain can worsen cognitive function, lead to depressive symptoms, reduce quality of life and trigger or exacerbate behavioural and psychological symptoms of dementia (BPSD) [6, 9, 19, 20]. The aetiology of BPSD is often multifactorial [21], however, pain is one of the most important causal factors for BPSD [6]. Indeed interventions targeting pain have been shown to be effective at reducing BPSD [22]. A person with advanced dementia may not either understand or be able to effectively communicate pain and so pain may be expressed through behaviours such as agitation or aggression [23]. In addition to the distress and discomfort untreated pain can cause, not correctly identifying pain as a trigger for these behaviours can lead to the inappropriate prescribing of potentially harmful psychoactive medications to people with dementia. In one study the presence of pain in nursing home residents was found to be significantly associated with the use of antipsychotic medication [24]. In addition to the harmful side effects of psychotropic medication in people with dementia [25, 26] these drugs often have a sedative effect which can mask behaviours that are indicative of pain [27], further contributing to under-diagnosis.

The vast majority of people living with dementia either live at home in the community or in a residential care setting such as a nursing home [28]. The general practitioner (GP) is the key healthcare professional for a person with dementia as they provide care in both the community and in nursing home settings. GPs play a pivotal role in assessing and managing pain in people with dementia, however, they are challenged by many aspects of dementia care including the management of BPSD [29–32] and providing end of life care [33]. Both the management of BPSD and the provision of end of life care in dementia require GPs to assess for and manage any underlying pain. In a recent qualitative study conducted by the authors, GPs reported difficulty identifying pain as a potential trigger for BPSD [34]. However, to the best of our knowledge no research to date has explored GPs’ perspectives on pain management in dementia. The aim of this study was to explore the knowledge and attitudes of Irish general practitioners to the identification and management of pain in people with dementia.

## Methods

An anonymous postal questionnaire was sent to a census sample of all GPs in Cork city and county in the southern region of Ireland in May 2017. Ethical approval was granted by the Social Research and Ethics Committee in University College Cork (Log2016–050).

### Questionnaire

There was no previously validated questionnaire available to address this research question. However, we did identify an appropriate questionnaire that was used in a previous study that explored nurses’ knowledge of and attitudes to pain management in dementia [35]. With the original authors’ permission we adapted this questionnaire for use with GPs. We (AJ, ML, TF), all practicing GPs with an academic interest in dementia care, reviewed the questionnaire to ensure its appropriateness and relevance to a general practice setting. Furthermore, the questionnaire was piloted with five GPs, all of whom have experience managing people with dementia in community and nursing home settings. Subsequently, minor amendments were made to enhance the clarity of the questionnaire. The final questionnaire developed for use in this study contained three sections. The aim of the first section was to capture demographic information from the GPs. The second section consisted of a series of five-point Likert-type statements exploring GPs’ knowledge and attitudes to pain in people with dementia. The final section provided GPs with the opportunity to give free-text responses. (See Additional file 1 for questionnaire).

### Sample

The questionnaire was posted with an information sheet to all 320 GPs practicing in the Cork region. With a large urban and rural population, a census sample of Cork GPs is largely representative of Irish GPs nationally. The sample size was calculated based on this census population of 320 with desired precision estimates of +/− 5% around a prior estimate of 50%. Based on these calculations to adequately power the study the sample size required was 175 respondents. All GPs were identified from the Irish Medical Directory [36]. A stamped addressed envelope was provided to return the questionnaire by post if the GP was willing to participate.

### Analysis

The data from the responses were coded and SPSS version 25 was used for statistical analysis. Chi-square tests and Mann-Whitney test were used to explore associations between demographic data and responses, with differences at a level of 95% probability and above regarded as statistically significant. The following demographic associations were explored; years in practice, presence of a nursing home commitment and the extent of the GPs nursing home commitment (as indicated by the number of residents the GP cared for). Free-text responses were entered into MS Word and were thematically analysed [37] by two of the authors (AJ,TF).

## Results

### Sample

Of 320 questionnaires sent, a total of 157 completed questionnaires were received, representing a response rate of 49% (157/320). The respondents had a broad mix of practice locations and years of experience. The demographic characteristics of respondents is displayed in Table 1.

**Table 1:** Participant demographics

Participant Demographics	N (%)
Practice Location	
City	45 (28.6)
Town	42 (26.7)
Rural	23 (14.6)
Mixed	47 (29.9)
Years of GP Experience	
0–5 yrs	20 (12.7)
6–15 yrs	48 (30.6)
16–25 yrs	41 (26.1)
> = 26 yrs	48 (30.6)
Nursing Home Commitment	
No commitment	49 (31.2)
Attends 1 nursing home	42 (26.5)
Attends 2 nursing homes	39 (24.8)
Attends 3 or more nursing homes	27 (17.2)

### Provision of care

Over two-thirds (108/157) of respondents had a nursing home commitment (mean number of nursing homes +/− SD = 1.35 +/− 1.19; range (0–5)). These GPs provided care to a total of 2393 people in nursing homes (mean number of patients = 15; range (1–112)). The respondents reported that just over half of these nursing home patients (1242/2393) had dementia. Of the GPs who provided care to nursing home residents, the majority (60.2%) did a regular round in the nursing home. Over half of the GPs with a nursing home commitment did 1 or more nursing home round per week (mean number of rounds per week = 1, range (0–5)). Rural GPs were significantly more likely to have a nursing home commitment (*P*–value = 0.042). There was no association found between the numbers of years a GP was in practice and having a nursing home commitment.

### Pain assessment in dementia

The overwhelming majority of GPs (98%) agreed that the presence of dementia can make pain difficult to assess (Table 2). A smaller majority of respondents (68.7%) felt that pain was under-recognised in patients with dementia. The majority of GPs surveyed agreed that observing behavioural and physiological indicators of pain and obtaining surrogate reports are important when assessing pain in a person with dementia. However, most GPs were unfamiliar with dementia-specific pain assessment tools with only 10% reporting any knowledge of their existence. The larger the nursing home commitment of the GP, as indicated by the number of nursing home residents they cared for, the more likely they were to be familiar with pain assessment tools for people with dementia (*P*-value = 0.048). However, of the respondents with a nursing home commitment only 14% were aware of guidelines/policies on pain management in the nursing homes they attended. The numbers of years the GP was in practice was not associated with an increased familiarity with pain assessment tools. Despite the lack of awareness of pain assessment tools, the majority (73.2%) of respondents believed that a pain assessment tool would be useful to increase recognition of pain in patients with dementia in nursing home settings.

**Table 2:** Responses to Likert statements on assessment of pain in people with dementia

Statement on assessment of pain in people with dementia	Agree ^a^ N, (%)	Neither agree nor disagree N, (%)	Disagree^a^ N, (%)
The presence of dementia can make pain assessment difficult.	154 (98.0)	3 (1.9)	0
A person with dementia is not able to accurately provide a self-report of their pain.	113 (72.0)	20 (14.0)	24 (15.2)
Pain assessment tools used for cognitively intact people are not appropriate for people with dementia.	103 (65.6)	33 (21.0)	21 (13.3)
I am familiar with pain assessment tools specifically available for use with a person with dementia.	16 (10.1)	20 (12.7)	121 (77.0)
When assessing pain in a resident with dementia, it is important to observe behavioural indicators of pain (e.g. facial expressions, body movements, posture).	154 (98.0)	2 (1.2)	1 (0.6)
When assessing pain in a resident with dementia, it is important to consider physiological indicators of pain (e.g. heart rate, blood pressure, temperature).	144 (91.7)	12 (7.6)	1 (0.6)
When assessing pain in a resident with dementia, it is important to consider a family/care givers report	150 (95.5)	7 (5.5)	0

^a^Note: The original Likert scale options “strongly agree” and “agree” were combined to “agree”, whereas the options “strongly disagree” and “disagree” were combined to “disagree”

### Management of pain in dementia

The responding GPs appeared less certain about aspects of the management of pain in dementia (Table 3). The overwhelming majority (95%) agreed that the treatment of pain should follow a step-wise approach. However, 26% were either disagreed or neither agreed nor disagreed that optimal treatment of pain is achieved when analgesics are given on a regular basis. The respondents were particularly uncertain about the safety of using opioid medications in people with dementia. Just over half of GPs surveyed (51.6%) agreed with the statement that opioid analgesics are safe to use when treating pain in dementia, while 34.4% neither agreed nor disagreed with the statement and 14% believed opioids were unsafe in this patient group. There was no statistically significant association found between the GP’s knowledge of the management of pain in dementia and their number of years in practice or the extent of their nursing home commitment.

**Table 3:** Responses to Likert-type statements on management of pain in people with dementia

Statement on the management of pain in people with dementia	Agree ^a^ N, (%)	Neither agree nor disagree N, (%)	Disagree^a^ N, (%)
Residents with dementia who are experiencing pain should be managed differently to cognitively intact residents.	52 (33.0)	34 (21.6)	71 (45.2)
The drug treatment of pain in a resident with dementia should follow a step-wise approach.	149 (94.9)	5 (3.1)	3 (1.9)
Optimal treatment of pain is achieved when analgesics are given on a regular basis.	116 (73.8)	31 (19.7)	10 (6.3)
Paracetamol is the best analgesic to use for residents with dementia who are experiencing chronic pain.	98 (62.4)	43 (27.3)	16 (10.2)
It is safe to use opioid analgesia to treat pain in residents with dementia.	81 (51.6)	54 (34.4)	22 (14.0)
Residents with dementia are less likely to become addicted to opioid analgesics than cognitively intact patients.	21 (13.3)	65 (41.4)	71 (45.2)
There is a greater risk of side effects from opioid analgesics (e.g. respiratory depression, confusion) when used in residents with dementia.	101 (64.3)	36 (22.9)	20 (12.7)
Non-drug based methods of pain control (e.g. TENs, Heat/Cold, massage, complimentary therapy) are useful in the management of pain in residents with dementia.	129 (82.1)	20 (12.7)	8 (5.09)

^a^Note: The original Likert scale options “strongly agree” and “agree” were combined to “agree”, whereas the options “strongly disagree” and “disagree” were combined to “disagree”

### Free-text responses

Of the 157 respondents, 49 GPs (31% of respondents) provided additional qualitative feedback in the free text section of the questionnaire. There were a number of themes identified from the free-text responses. The three main themes identified were; (i) the role of pain assessment tools, (ii) the importance of input from carergivers and (iii) challenges of resource limitations. Verbatim quotations are presented here to illustrate the themes. [38] These particular quotations were selected as they were considered to be typical of the responses that underpinned the development of each theme.

#### Theme 1: Role of pain assessment tools

Several of the responding GPs expressed a desire for guidance in the area of pain assessment and management in dementia:

“*Would love guidance on pain management in these patients” (Respondent_79, experienced GP, urban practice, no nursing home commitment)*

Some respondents expressed apprehension about introducing pain assessment tools.

*“I feel a lot of ‘tools’ can lead to unnecessary work if arbitrarily based on BP readings, heart rates etc e.g. MEWS score in hospital which is why I’d be wary of their use. Would need to be used judiciously”*

*(Respondent_84, recently qualified GP, rural practice, with nursing home commitment)*

Many GPs perceived pain assessment tools as yet another tool that would add to GPs’ workload without necessarily improving care.

*“A pain assessment tool wouldn’t be helpful in nursing homes as it would add more workload to already onerous paperwork”*

*(Respondent_128, recently qualified GP, urban practice, with nursing home commitment)*

#### Theme 2: The importance of input from caregivers

A second theme identified was the value GPs placed on the input of relevant caregivers, who knew the person with dementia well, when assessing pain:

*“Key component to good assessment depends on good collateral history - family/close friends, nursing staff/carers.”*

*(Respondent_46, experienced GP, rural practice, with a nursing home commitment)*

In particular many GPs highlighted the important role of nurses in pain assessment:

*“Feedback from nurses helps to make appropriate prudent descriptions.”*

*(Respondent_75, experienced GP, mixed practice setting, with a nursing home commitment)*

The GPs described how they relied on the nursing staff and trusted their opinion when assessing a person with dementia:

*“An experienced nurse is the best person to rely on, they know as do the carers – listen to them and you’ll get it right.”*

*(Respondent_140, experienced GP, urban practice, with a nursing home commitment)*

#### Theme 3: The barriers of under-resourcing

A third theme identified was the challenges GPs experienced providing care to people with dementia given the current underfunding of Irish general practice.

*“Dementia [is] not currently a paid ‘chronic disease’ in GP, poor remuneration.”*

*(Respondent_114, mid-career GP, mixed practice setting, no nursing home commitment)*

The GPs reported extensively about the impact of recent austerity cuts in Ireland which dramatically cut funding to GPs who provide care to nursing home residents:

*“Usual gripe – fee for attending nursing home residents in now 1/3 of what it was in 2008! Hhhmmmm…..”*

*(Respondent_70, mid-career GP, rural practice, with a nursing home commitment)*

Several GPs also stated that they no longer provided care to nursing home patients as a result of these reductions in remunerations:

*“I have given up nursing home care. Funding poor. Bureaucracy a problem.”*

*(Respondent_26, experienced GP, urban practice, no nursing home commitment)*

## Discussion

This is the first study that has explored GPs’ assessment and management of pain in people with dementia. Our findings suggest that GPs are confident in many aspects of assessing pain in people with dementia such as the value of observing behavioural and physiological indicators and the importance of surrogate reports but that they are challenged by many aspects of assessing and managing pain in dementia. The majority of GPs surveyed believed that a person with dementia cannot self-report pain and the vast majority of GPs were unfamiliar with dementia-specific pain assessment tools. In the absence of either a self-report or a standardized observational tool to assess pain, the responding GPs appeared to rely significantly on surrogate reports from family members and nursing home staff when assessing pain in dementia. Although the majority of responding GPs welcomed the idea of guidance in the area of pain assessment and management in dementia, in the free-text comments many questioned the value of a standardized, observational pain tool. Furthermore, when managing pain in people with dementia the GPs were particularly uncertain about the role of opioid medication and the consequences of the use of opioids in people with dementia. While the majority of GPs agreed that in order to achieve optimum pain relief analgesia should be prescribed regularly, over a quarter of GPs surveyed did not agree with this statement. The inference being that these GPs are favouring ‘as required’ analgesic medication, a sub-optimal method of pain control. Although we did find that GPs with a larger nursing home commitment were more likely to be familiar with dementia-specific pain assessment tools, in general, the experience level of the GP was not associated with increased levels of knowledge or a more positive attitude towards pain assessment and management in dementia.

### Comparison with existing literature

Our findings indicate that GPs’ value good communication with family members and nursing home staff when managing pain in people with dementia. This finding is similar to previous studies with community pharmacists [10] and nurses [35]. In previous research on GPs’ educational needs in dementia [29] both GPs and family caregivers emphasized the importance of good channels of communication in dementia care. In our study, the GP respondents also emphasized the importance of a report from nursing staff or family carer when assessing pain in the qualitative free-text responses. In our study nursing staff were seen by the GPs to facilitate pain assessment. This is in contrast to findings from a previous study that examined nurses knowledge and attitudes to pain management in dementia where nurses identified a lack of GP support as a barrier to successful pain management [35]. Previous research with GPs found that consistency of care was an important factor in improving relationships between GPs and nursing staff [34]. In that study structured visits by the GP to the nursing home were seen to facilitate the provision of continuity of care and led to good communication channels between the GP and nurses [34]. Effective communication between nursing staff and GPs is an essential component of any optimisation of pain assessment and management in nursing home settings.

The majority of respondents in our study believed that people with dementia could not accurately provide a self-report of pain. However, self-reporting of pain is considered the gold standard method of pain assessment [39] and can be a reliable way of assessing pain in people with dementia [39–41]. Best practice recommendations advise that where possible attempts should always be made to elicit self-reports of pain from the person with dementia [39]. Although in the very advanced stages of dementia many individuals may be unable to self-report pain [14], people with mild-moderate [24, 42] and in some cases severe dementia [43] have been found to provide valid self-reports of pain. Our finding echoes previous research with nursing home managers which found that only 8.3% of respondents felt that people with dementia could self-report pain [44]. The large majority of GP respondents agreed with the statement that *‘a person with dementia is not able to provide a self-report of pain’.* This finding could mean that an attempt is not being made to elicit a self-report from a person with dementia. However, this needs to be explored further, ideally with qualitative research, to establish the impact this attitude has on how GPs assess pain in people with dementia.

Nearly all GP respondents agreed that patient observation was a critical part of pain assessment in dementia. Despite this belief the vast majority of respondents were not using any validated, standardised, observational approach. Observational methods are central to clinical assessment, especially when a person lacks the ability to self-report, however, there is a risk of observer bias if there is no standardized approach to the observation [40]. A large array of pain assessment tools exist for use in people with dementia - a recent systematic review of pain assessment tools included 28 such tools [45]. However, the vast majority of GP respondents were unaware of these tools. Similar to previous findings from a study with community pharmacists [10], the more experience the GP had with dementia the more likely he or she was to be aware of these tools. The lack of awareness of dementia-specific pain assessment tools is a particularly noteworthy finding since the majority of respondents thought that having such a pain assessment tool would be helpful. This highlights an incongruity between research in this area and real-life clinical practice. These tools appear to be rarely used in general practice.

The responding GPs lack of familiarity with pain assessment tools may be surprising to researchers in the area but may be unsurprising to front-line GPs. GPs do not readily embrace assessment tools [46, 47]. They are inductively trained to rely on their clinical skills. This is in contrast to nursing staff who are specifically trained to use and rely on assessment tools. In the free-text responses some GPs feared the additional workload such tools could bring and questioned what clinical value they would add. Similarly, recent qualitative research exploring GPs’, hospital physicians’ and nurses’ perspectives on the use of observational pain tools in people with dementia identified a number of barriers to using observational pain tools, one being the perceived lack of value in using them [48]. Furthermore, participants in that qualitative study described using the pain tools to comply with local recommendations but not actually using the results to inform treatment decisions [48]. This echoes some of the concerns raised by participants in our study that a pain tool would become another source of paper-work rather than a tool to aid clinical decision making. GPs are not usually the healthcare professional tasked with completing a pain assessment tool; in a nursing home setting that role would typically fall to the nursing staff. GPs do still need to be aware of these tools in order to interpret the findings and generate appropriate management plans in discussions with nursing staff. However, clinicians and nurses can find the results of pain assessment tools difficult to interpret [49]. Implementing these observational pain assessment tools in isolation, without adequate guidance on how to interpret the results, will not lead to improved treatment of pain [14]. The tools in themselves will not result in improved care unless they are combined with guidance that will help clinicians to translate a pain score into an appropriate treatment plan.

Respondents appeared unsure about the safety of opioid analgesics in people with dementia. This is similar to previous research conducted with nurses [35], nurse managers [44] and community pharmacists [10] all of whom had similar concerns regarding the safety of prescribing opioids to people with dementia. Guidelines on pain management in the older adult do recommend considering opioid analgesics for patients with moderate to severe pain, particularly if the pain is causing functional impairment or reducing quality of life [3]. The uncertainty the GP respondents felt regarding the safety of opioid medication in dementia is perhaps understandable; prescribing opioids to an older adult is not without its complexities. Age is a significant predictor of opioid related harm [50, 51]. Adverse effects of opioids can include respiratory depression, sedation, constipation, nausea and dizziness [52]. Many of these adverse effects increase with age [53] and frailty [51] both of which are associated with dementia. Furthermore, these adverse effects can be particularly problematic to identify in a person with advanced dementia because of their reduced ability to communicate. However, when managing pain in people with dementia the adverse effects of opioids needs to be weighed up against the harmful effects of undertreating pain. Although a narrow majority agreed with the statement that opioids were ‘*safe in people with dementia’*, a larger majority of respondents agreed with the statement that there is *‘a greater risk of side effects from opioid analgesics when used in people with dementia’.* Many respondents appeared to feel that, despite the increased risks, the use of opioids in people with dementia was still “safe”. How this belief influences the GP’s prescribing is unclear. Previous research suggested that people with dementia may receive less opioids [54]. A qualitative study which examined GPs’ perspectives on prescribing opioids for chronic pain found that fear of causing harm was a barrier to prescribing opioids to the older adult [55]. Despite this a recent systematic review identified that, internationally, prescribing of opioids to nursing home residents has increased over time [56]. Similarly, a large Danish study found that buprenorphine and fentanyl patches were more commonly prescribed to people with dementia [57]. It is possible that these opioid patches are perceived as having less side effects than oral opioids or as being more tolerated by people with dementia. However, these transdermal opioid patches typically contain a stronger opioid dose than oral opioids, and since the adverse effects of opioids are dose related these patches are likely to result in more, not less, side effects. Prescribers’ perspectives of the role of different opioid medications in the management of pain in dementia is an important area that could be explored in future research.

Although optimal treatment of pain is achieved when analgesics are given on a regular basis [58], over a quarter of respondents in our study did not agree with this statement. A similar finding was reported in previous research with nurses [44] where 20% of respondents either agreed, or neither agreed nor disagreed, with a statement that ‘optimal pain treatment of pain relief is achieved when analgesics are given in a PRN (or as required) way’. If the value of regular administration of analgesia is not recognized this could result in the prescribing of analgesics in the less-effective ‘when required’ way. Research conducted in nursing homes in Northern Ireland in 2015 found that in the majority of residents with dementia, analgesic medication was prescribed ‘when required’ and not regularly [24]. Likewise, research from the U.S. found that cognitively impaired nursing home residents were less likely than their cognitively intact peers to be prescribed regular, scheduled analgesia and were more likely to be prescribed analgesic medication in an ‘as required’ way [18]. To receive this ‘as required’ medication a person would either need to ask the nurse for pain relief or the nurse would need to identify that the person was in pain and give them pain relief [18]. In the case of a person with advanced dementia neither of these situations are very likely to occur. In view of their cognitive and communication difficulties a person with advanced dementia is unlikely to self-request analgesia and we know from existing evidence that there are a number of barriers to nurses identifying and initiating pain relief in a person with dementia [35, 44].

### Areas for future research & implications for policy & practice

This study highlights several areas of GPs assessment and management of pain in dementia that could be explored with future research. There were some findings that warrant further exploration in future research such as the use of ‘as required’ medication and the belief that people with dementia cannot self-report pain. Future qualitative research with GPs in this area would help gain a deeper understanding of the context and nuances that are involved in this complex area. It would be important to ascertain how GPs’ knowledge of and attitudes towards pain in people with dementia impacts on their actual prescribing of analgesia to people with dementia. A quantitative study of current prescribing of analgesia to people with dementia both in nursing homes and in the community would be an important complementary study. These studies would lead to a more extensive understanding of the problem and would help to inform the development of effective interventions to improve the management of pain in dementia in primary care settings.

There is a role for educational interventions for GPs that focus on pain assessment and management in dementia. The results of this current study will inform the ongoing, national roll-out of educational interventions for GPs in dementia care that have being developed by our research team [59, 60] as part of the implementation of the Irish National Dementia strategy [61]. Previous educational interventions for GPs in the area of pain management in dementia have used teleconferencing technology based on the Project ECHO© model and have been found to improve healthcare professionals’ knowledge and self-efficacy of pain assessment and management in advanced dementia [62]. Such educational interventions would be particularly acceptable to general practitioners as they eliminate the need for travel and don’t require any prolonged periods away from practice. Another important finding was in relation to educational initiatives was that GPs see nurses as facilitators to optimum pain management, whereas in previous studies [35, 44] nurses identified GPs as barriers to optimum pain management. This highlights a communication gap between these two professional groups, both of whom play a pivotal and complementary role in the assessment and management of pain in dementia. This finding underlines the importance of inter-professional educational initiatives in this area. Palliative care is another professional group that have a significant role to play in the management of pain in dementia, particularly in complex cases, yet we know that many people with dementia are not routinely assessed to determine their palliative care needs [63]. An inter-professional educational approach including all the relevant professional groups could help improve communication and bridge professional divides [64], which would play an important role in improving the care provided to people with dementia who are living with chronic pain.

Although relevant and important we know that educational interventions alone have limited effect in changing GPs’ behaviour in dementia care [65, 66]. To effectively improve GPs’ performance in dementia care, education needs to be combined with adequate reimbursement and organisational incentives [67]. A previous systematic review highlighted how time constraints and inadequate remuneration act as barriers to optimum diagnosis and management of dementia in primary care [68]. In Ireland general practice receives only 4.5% of the overall health budget, significantly lower than other European countries [69]. Additionally, recent government-led austerity cuts in Ireland have dramatically cut funding to general practice, these cuts have particularly affected general practitioners providing care to nursing home residents. In our study the dissatisfaction of GPs with the current under-resourcing of dementia care was evident in the free-text responses. Many GPs discussed the challenges of providing optimum care to people with dementia in the context of current resource limitations, reporting that they can no longer provide care to nursing home residents. Like many other European countries [70–72], Irish general practice is currently facing a recruitment crisis [73]. This recruitment crisis is particularly affecting rural general practice, therefore, it was notable that rural based GPs were significantly more likely to provide care to nursing home residents than non-rural GPs. In the face of this recruitment crisis and in the context of current inadequate reimbursement the future of GP led nursing home care is uncertain. Future policy needs to focus on adequate resourcing of dementia care in both community and nursing home settings.

The study results have several immediate practical implications for GPs caring for people with dementia. The implications for GPs assessing pain in people with dementia include; being more aware of the increased risk of pain in people with dementia, considering pain as a potentially reversible trigger for BPSD, providing people with dementia an opportunity to self-report pain and familiarising themselves with the pain assessment tools that may already be in place in the nursing homes they attend in order to facilitate more effective communication with the nursing home staff. From a pain management perspective one practical implication for GPs is that if pain is identified, or suspected, then it is important that the person’s pain is not under-treated. Inappropriate prescribing in people with dementia does not just mean over-prescribing. It also pertains to under-prescribing of appropriate medications that can improve comfort and overall quality of life.

### Strengths and limitations

Although a 49% response rate is modest, it is typical of postal surveys with this professional group [74]. Our response rate is similar to the response rate in a previous national study on GPs attitudes to diagnosis in dementia [75] and significantly higher than a recent online survey of Irish GPs referral patterns in dementia care [76]. To adequately power this study a sample size of 175 respondents was required. Therefore, with 157 respondents the study was marginally underpowered. Furthermore, this study was a census study of a specific geographical area in the southern region of the Republic of Ireland. This may affect the generalisability of the study, however, the respondents’ demographic characteristics, in terms of years of experience and practice location, are representative of Irish GPs nationally [77]. A large proportion of respondents had a nursing home commitment. There is currently no Irish data available on the number of GPs who attend nursing homes, therefore, it is difficult to ascertain whether this represents a respondent bias. It is possible that GPs with a nursing home commitment were more likely to respond to the survey as they may have been more interested in the research topic. Finally, this was a cross-sectional survey of self-reported knowledge and there is evidence to suggest that when self-reporting physicians may underestimate their knowledge in an area [78]. The nuances of clinically complex areas such as this are difficulty to fully address with a single study that rely primarily on self-reporting measures. However, since there is very little research exploring GPs experiences of managing pain in people with dementia our chosen methodological approach is a necessary and reasonable place to start.

## Conclusions

Despite the pivotal role GPs play in dementia care their experience of managing pain in dementia is greatly under-researched. Prior to this study very little was known about GPs’ knowledge of and attitudes towards the assessment and management of pain in dementia. This study enriches existing literature in the area of pain management in dementia care and also raises some important issues that should be explored in future research. Pain management in a person with dementia touches on some of the most fundamental aspects of a GP’s role as a patient advocate. It is, therefore, challenging to identify aspects of care that could, perhaps, be improved upon. However, without identifying these areas we cannot design effective interventions to appropriately address them. The results of this study will be used to inform the development of interventions to improve the management of dementia in general practice as part of the wider implementation of the Irish National Dementia Strategy.

## Additional file

Additional file 1: Questionnaire A blank copy of the questionnaire (DOCX 19 kb)

## Abbreviations

BPSD: Behavioural and psychological symptoms of dementia
GPs: General practitioners
