# Supplementary material for: The knowledge and attitudes of general practitioners to the assessment and management of pain in people with dementia
Source: BMC Fam Pract. 2018 Oct 10;19:166. doi: 10.1186/s12875-018-0853-z (PMC6178252; doi:10.1186/s12875-018-0853-z)
Supplement: Supplementary file 1 — Questionnaire A blank copy of the questionnaire (DOCX 19 kb) [file 12875_2018_853_MOESM1_ESM.docx]

**Additional file 1 – The questionnaire**

**Section A: General Information**

This section is concerned with gathering information about you and where you work.

1. Where is your practice based? City ☐ Town ☐ Rural ☐ Mixed ☐
2. How many years are you practicing as a GP?

0-5 ☐ 6-15☐ 16 -25☐ 26+ ☐

1. Do you provide regular care to residents of nursing homes? Yes ☐ No☐

*(if answer to Q3 is “no” then please skip to Q8)*

1. How many nursing homes do you regularly attend? __________
2. How many nursing home residents do you look after? __________
   1. Approx. how many of these residents suffer from dementia? ________
3. Do you do regular visits (e.g. weekly round) to nursing homes?

Yes☐ No☐

- 1. How many rounds do you do per week? _________________

1. Are there guidelines/policies on pain management in the nursing home? Yes ☐ No☐ Don’t know☐
2. Do you think that pain is under-recognised in patients with dementia? Yes☐ No☐ Don’t know☐
3. Do you think that a pain assessment tool, for the recognition of pain in patients with dementia, would be helpful in Nursing Homes?

Yes☐ No☐ Don’t know☐

**Section B: Assessment and Management of Pain in Dementia**

This section of the questionnaire is divided in to two parts, each of which is concerned with a different aspect of pain in residents with dementia. For each statement please indicate with a tick how strongly you agree or disagree using the scale provided.

| **ASSESSMENT OF PAIN IN RESIDENTS WITH DEMENTIA** | | | | | |
| --- | --- | --- | --- | --- | --- |
| ***Statement*** | ***Strongly Agree*** | ***Agree*** | ***Neither Agree nor Disagree*** | ***Disagree*** | ***Strongly Disagree*** |
| 1. The presence of dementia in a person can make pain assessment difficult. |  |  |  |  |  |
| 1. A person with dementia is not able to accurately provide a self-report of their pain. |  |  |  |  |  |
| 1. Pain assessment tools used for cognitively intact residents are not appropriate for people with dementia. |  |  |  |  |  |
| 1. I am familiar with pain assessment tools specifically available for use with a person with dementia. |  |  |  |  |  |
| 1. When assessing pain in a resident with dementia, it is important to observe behavioural indicators of pain (e.g. facial expressions, body movements, posture). |  |  |  |  |  |
| ***Statement*** | ***Strongly Agree*** | ***Agree*** | ***Neither Agree nor Disagree*** | ***Disagree*** | ***Strongly Disagree*** |
| 1. When assessing pain in a resident with dementia, it is important to consider physiological indicators of pain (e.g. heart rate, blood pressure, temperature). |  |  |  |  |  |
| 1. When assessing pain in a resident with dementia, it is important to consider a family/care givers report. |  |  |  |  |  |
| **MANAGEMENT AND TREATMENT OF PAIN IN RESIDENTS WITH DEMENTIA** | | | | | |
| 1. People with dementia who are experiencing pain should be managed differently to people who are cognitively intact. |  |  |  |  |  |
| 1. The drug treatment of pain in a person with dementia should follow a step-wise approach. |  |  |  |  |  |
| 1. Optimal treatment of pain is achieved when analgesics are given on a regular basis. |  |  |  |  |  |
| 1. Paracetamol is the best analgesic to use for people with dementia who are experiencing chronic pain. |  |  |  |  |  |
| 1. It is safe to use opioid analgesia to treat pain in people with dementia. |  |  |  |  |  |
| 1. People with dementia are less likely to become addicted to opioid analgesics than cognitively intact patients. |  |  |  |  |  |
| ***Statement*** | ***Strongly Agree*** | ***Agree*** | ***Neither Agree nor Disagree*** | ***Disagree*** | ***Strongly Disagree*** |
| 1. There is a greater risk of side effects from opioid analgesics (e.g. respiratory depression, confusion) when used in people with dementia. |  |  |  |  |  |
| 1. Non-drug based methods of pain control (e.g. TENs, Heat/Cold, massage, complimentary therapy) are useful in the management of pain in people with dementia. |  |  |  |  |  |

**Have you any further comments or remarks to make in relation to this questionnaire or the subject of pain in patients with dementia?**

|  |
| --- |

**Thank you for your time!**
